# Comparison of Faunal Scavenging of Submerged Carrion in Two Seasons at a Depth of 170 m, in the Strait of Georgia, British Columbia

**DOI:** 10.3390/insects8010033

**Published:** 2017-03-13

**Authors:** Gail S. Anderson, Lynne S. Bell

**Affiliations:** Centre for Forensic Research, School of Criminology, Simon Fraser University, 8888 University Dr., Burnaby, BC V5A 1S6, Canada; lynneb@sfu.ca

**Keywords:** submergence, marine, amphipods, taphonomy, forensic, arthropods, underwater cabled laboratory

## Abstract

The taphonomy of carcasses submerged in the ocean is little understood, yet it is extremely important ecologically and forensically. The objectives of this study were to determine the fate of pig carcasses as human proxies in the Strait of Georgia at 170 m in spring and fall. Using Ocean Networks Canada’s Victoria Experimental Network Underseas (VENUS) observatory, two carcasses per season were placed under a cabled platform hosting a webcam and instruments measuring water chemistry. Two minutes of video were recorded every 15 min. In spring, Lyssianassidae amphipods and *Pandalus platyceros* were immediately attracted and fed on the carcasses, the amphipods removed the bulk of the soft tissue from the inside whilst the shrimp shredded the skin and tissue. The carcasses were skeletonized on Days 8 and 10. In fall, *Metacarcinus magister* was the major scavenger, removing most of the soft tissue from one carcass. Amphipods did not arrive in large numbers until Day 15, when they skeletonized the scavenged carcass by Day 22 and the less scavenged carcass by Day 24. Amphipods remained for some days after skeletonization. This skeletonization was very different from previous experiments at different depths and habitats. Such data are very valuable for predicting preservation, planning recoveries, and managing family expectations.

## 1. Introduction

The decomposition and insect colonization of carrion is well understood in the terrestrial environment and has been studied in a variety of habitats and geographic regions, globally [1,2], both for ecological value [3] and for medico-legal application or forensic entomology casework [4]. However, the taphonomy of carrion in the aquatic world is much less studied. Some studies and case reviews have been conducted in freshwater environments [5,6,7,8,9,10]. Conducting carrion research in water is much more difficult than on land due to accessibility and safety issues; as well, changes in the physical and chemical environment can occur over just a few meters [11,12]. The marine environment is particularly challenging as it is more complicated, expensive, and difficult to place and monitor carcasses. Therefore, only a few studies have been conducted.

It is important to understand the impact of submergence on decomposition as human bodies are frequently recovered from the ocean, from natural disasters such as tsunamis, vessel sinking, and plane crashes as well as recreational and suicidal deaths, and homicide body disposals [13,14,15]. It is, therefore, important that we have a better understanding of the fate of a body in the ocean. Marine ecologists have studied whale falls and carcasses of other large marine animals for decades, at first studying already deceased carcasses, fortuitously discovered. Later studies have involved deliberately placing whales and other large carcasses on the sea floor, often at great depths [16,17]. Such enormous carcasses have been shown to go through a number of decompositional stages, some of which may take years to complete. Our knowledge of the fate of smaller carcasses, more akin to humans, is much more restricted and is mostly confined to anecdotal case reports of drowning victims [18,19,20,21].

Pig (*Sus scrofa* L. 1758) carcasses are frequently used as human proxies in decomposition studies as they are similar in size to the human torso, are relatively hairless, omnivorous, with similar skin [22]. In earlier work, pig carcasses were submerged at 7.5 and 15 m in the Howe Sound, which is a network of fjords with the mouth of the sound opening into the Strait of Georgia, British Columbia (BC). Valuable data were generated on the impact of season, depth, water, and seabed conditions on faunal colonization and carcass degradation [11,23], but observation times were limited by diver availability and weather conditions, limitations common to such work.

In 2006, Ocean Networks Canada’s (ONC) Victoria Experimental Network Underseas (VENUS) observatory was established. This is a cabled underwater laboratory which provides an unparalleled method for studying undersea taphonomy in real-time, with the ability to almost constantly observe carcasses, while continuously monitoring many environmental parameters. Pig carcasses were first deployed in Saanich Inlet, a deep water fjord, adjacent to Vancouver Island, BC which is hypoxic for much of the year. Under these conditions, carcasses were scavenged rather than decomposed, and in some conditions could remain intact for months [12]. This work continued into the much busier and more oxygenated waters of the Strait of Georgia at a depth of 300 m (VENUS Central Node) where pig carcasses were skeletonized by Lyssianassidae amphipods within 3–4 days, depending on the season. The current research extends this work into shallower waters in the Strait of Georgia at 170 m (VENUS East Node) and examines the impact of submergence in spring and fall on carcass decomposition and faunal scavenging.

## 2. Materials and Methods

This work was performed in collaboration with ONC’s VENUS observatory, a cabled underwater observatory based at the University of Victoria on Vancouver Island, BC.

The research sites were at a depth of 170 m in the Strait of Georgia (latitude: 49°02.569′ N, longitude: 123°19.032′ W), part of the Salish Sea. The Strait of Georgia is considered to be Canada’s most important marine waterway and is one of the busiest seaports in North America [24], shortly to become much busier. It is a large body of water between the mainland coast of BC, Vancouver Island and northern coastal Washington State.

The VENUS observatory includes shore stations which power communication and power hubs or nodes on the seafloor. Each node then connects to a science instrument interface module (SIIM) via fiber optic cable and, from there, to an array of oceanographic sensors, instruments, and cameras, which are continuously collecting physical, chemical, acoustic, and photographic data. The shore station also provides communication links between the instrument arrays and the University of Victoria, which houses a Network’s operation centre (NOC), which manages the functioning of the instruments and a data management and archive system (DMAS), which receives and processes the data which can then be accessed by researchers anywhere in the world via the Internet [24].

**Figure 1:**
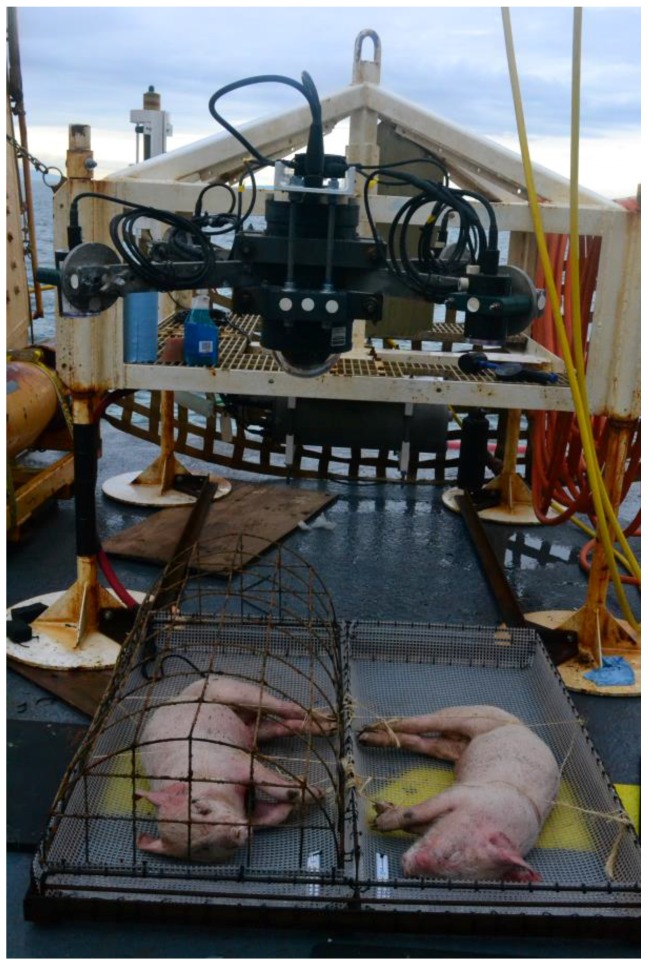
Two pig carcasses on the pig platform, attached to the instrument platform, ready to be deployed. A digital webcam was positioned above the carcasses. Frame, cage, and trays designed by Chris Sundstom and instrument platform designed by Paul Macoun, ONC, VENUS observatory.

In both spring (7 March 2014) and fall (20 September 2014), two paired, 16–23 kg freshly-killed pig carcasses were utilized. Simon Fraser University Animal Care Committee permission was obtained to purchase dead pigs, Animal Care Permission #1027CR-11. The pigs were humanely euthanized by a licensed butcher and were received immediately after death. They were not exsanguinated or frozen but were refrigerated on the research vessel prior to placement. They were refrigerated from leaving the dock until the vessel was at the research site and ready to deploy, (approximately eight to ten hours). The paired carcasses were placed onto a specially designed platform (designed by Chris Sundstom, ONC) attached to an instrument platform (designed by Paul Macoun, ONC) (Figure 1).

One carcass was completely exposed, while the other was protected by widely-spaced bars (14–15 cm × 10–12 cm) to conserve at least one carcass in the event that sharks fed on the remains, but still allow arthropod access. This design had been proven successful in previous experiments at 300 m in the Strait of Georgia [25]. The carcasses were positioned directly below a digital webcam (AXIS Q6034 720 p HD, Axis Communications, Lund, Sweden) with a lighting array (Figure 1). The camera was continuously powered on, but lights were timed to come on every 15 min for a two minute period during which the camera was programmed to pan over both carcasses and the surrounding area. The camera could also be manually controlled when needed.

The entire pig platform was connected by fiber optic cable to the nearest VENUS instrument platform (VIP) and, from there to the shore station. A number of instruments were deployed with the carcasses to measure oceanic conditions. These included an oxygen optode (SeaBird 43 Oxygen (S/N 1807 (spring) and Aanderaa Optode 4175C (S/N 1795) 23204 (fall) and a CTD (SeaBird 16 Plus 6935 and SeaCat SBE 16plus 4998) which recorded temperature (°C), salinity (psu), pressure (decibar), conductivity (S/m), density, as sigma T (kg/m^3^), and speed of sound (m/s) at per minute intervals. In the spring experiments, the instruments were placed on the VIP approximately 70 m from the carcasses and in fall they were placed close to the carcasses on the instrument platform attached to the pig platform.

The pigs were tied onto the platform with hemp as, although no bloat or refloat would occur at this depth [26], past experience has shown that animal activity can move a carcass out of camera view very rapidly [12,23]. The platforms were deployed from a research vessel to the seabed using the vessel’s crane, then positioned at the predefined site by a remotely-operated submersible vehicle (ROV). The ROV then connected the instruments and camera to the fibre optic cable, which connected to the VENUS array for remote monitoring. The day of submergence was deemed Day 0. Once deployed, there was no physical access to the carcasses. The platform and carcass remains were recovered approximately six months after deployment (Day 196 in spring and Day 191 in fall).

Videos and instrument data were downloaded from the VENUS website [24] for analysis. At first all videos were viewed, then videos were viewed every hour. Over time, viewing was reduced to every two hours, then to every 6 h, then gradually reduced to daily, and eventually every five days. At any time, if a dramatic change occurred between viewings, the intermediate videos were observed.

## 3. Results

Table 1 shows the major fauna which were attracted to the carcasses in spring and fall over the duration of the experiments.

### 3.1. Spring Faunal Scavenging

**Figure 2:**
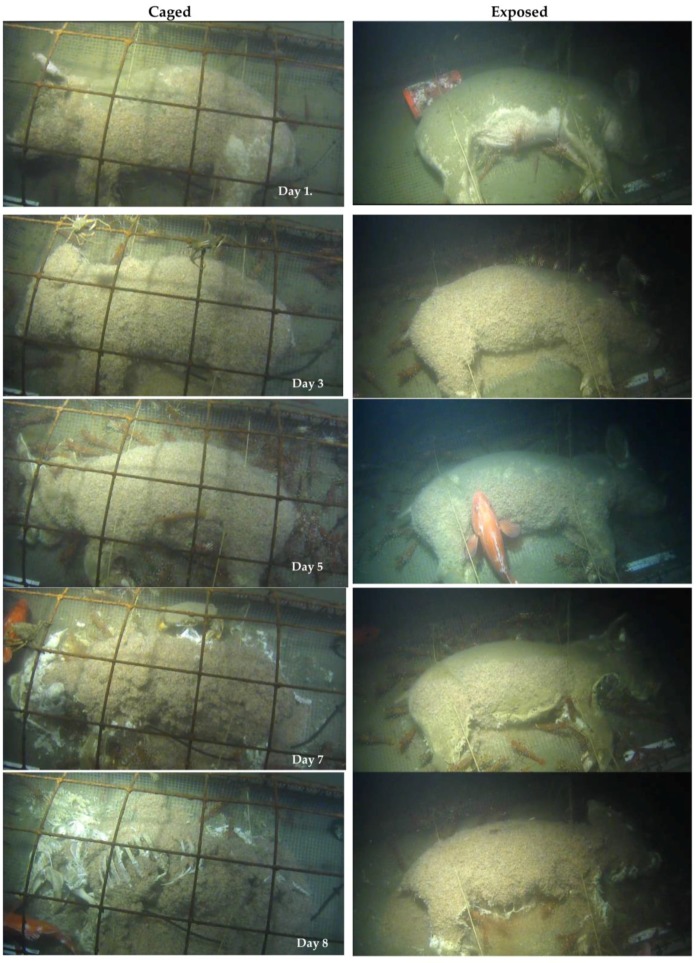
Faunal scavenging of caged and exposed carcasses in spring at 170 m in the Strait of Georgia (Ocean Network Canada’s VENUS observatory).

A few Lyssianassidae amphipods and gastropods were attracted to the exposed carcass within 2.5 h and began to settle a few hours later. Although not collected, earlier research suggests the amphipods are from the *Orchomene* complex, *Orchomenella* aff. *obtusa* (Sars, 1891) [25]. Although a few amphipods were attracted to the exposed carcass within hours, they arrived later to the caged carcass. A very strong current was visibly flowing over the carcasses which may have impacted the exposed carcass more than the caged carcass. By Day 1 in spring, many more amphipods had settled on the caged carcass and a few were beginning to settle on the exposed carcass (Figure 2). Several three spot shrimp, *Pandalus platyceros* Brandt, 1851, and other smaller *Pandalus* species were attracted to the carcasses.

By Day 2, the caged carcass was thickly covered in amphipods and, although fewer amphipods were present on the exposed carcass, the numbers gradually increased (Figure 2). Numbers of *P. platyceros* and other shrimp also increased. A single Dungeness crab, *Metacarcinus magister* (Dana, 1852) was present, as were several decorator crabs (superfamily Majoidea). The number of amphipods continued to increase daily and spread slightly onto the ropes and surrounding area. The numbers of *P. platyceros* also increased (Figure 2), and could be seen moving towards the carcasses across the surrounding substrate (Video S1). Both adult and juvenile shrimp were present and picked at the skin and anus, sometimes picking through layers of amphipods.

On occasions, a shortspine thornyhead fish (*Sebastolobus alascanus* Bean, 1890) swam through and brushed amphipods from the carcass to reveal undamaged skin beneath, indicating that the amphipods were not breaking through the skin (Figure 2). A few *M. magister* fed on the carcasses on occasion, but were rare and usually small. By Day 5, tissue loss was first apparent at the stomach area, under the layers of amphipods in both carcasses and, by Day 6, it was clear that areas with fewer amphipods and larger numbers of *P. platyceros*, such as the ears and legs, exhibited shredded skin and tissue, whereas the skin covered by amphipods was undamaged, with hair, and even silt on the skin, still visible. Instead, the amphipods entered the carcasses through orifices and scavenged areas, and removed tissue mass from the inside. By Day 7, the caged carcass had lost much of its biomass, with skin and tissue shredded by *P. platyceros* and occasional *M. magister* and internal tissue removed by amphipods (Figure 2). Leg bones were exposed, then almost separated from the carcass. The exposed carcass continued to support fewer amphipods and more *P. platyceros*, with resulting skin shredding and some mass loss (Figure 2). During Day 8, the caged carcass was completely skeletonized over the day, although still fully articulated with large numbers of amphipods covering the bones, as well as *P. platyceros* feeding on pieces of tissue. The numbers of shrimp decreased once the carcass was completely skeletonized but large numbers of amphipods remained.

Several *S. alascanus* continued to be present, usually just swimming over the carcasses, sometimes going after shrimp but were seen occasionally to take a bite at the tissue. The exposed carcass continued to lag behind but, over the day amphipods completely covered the entire carcass, spilling onto the cage floor and roosting on the ropes, possibly migrating from the depleted caged carcass. Tissue loss increased over the day and several sudden bursts of red/orange fluid suggested that the abdominal area was breached (Figure 2). As with the caged carcass, much of the exposed tissue was shredded by shrimp. The exposed carcass was partially skeletonized one day later than the caged carcass and completely skeletonized with bones disarticulated by Day 10. Despite skeletonization, large masses of amphipods remained on the bones and in the cages until Day 12 with a few *P. platyceros* and persistent *S. alascanus*, which occasionally disarticulated and ate bones (Video S2). The majority of the cartilage was removed by Day 13. Many gastropods appeared on the bones for a few days, and at least one, and up to five, *S. alascanus* were present almost continuously until recovery. On Days 103–105, a pair of *M. magister* were clasped together on the cage as the male waited for the female to shed and then mate.

### 3.2. Fall Faunal Scavenging

**Figure 3:**
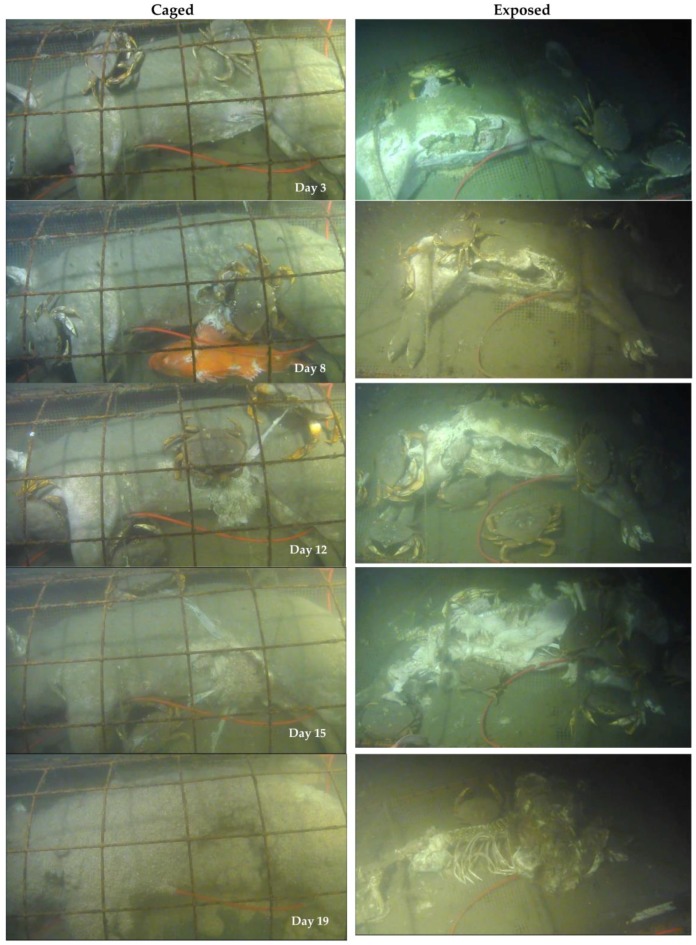
Faunal scavenging of caged and exposed carcasses in fall at 170 m in the Strait of Georgia (Ocean Network Canada’s VENUS observatory).

Fall carcasses were scavenged much differently than spring carcasses with almost no amphipod activity until Day 15 and almost no shrimp, but large numbers of *M. magister* (Table 1, Figure 3). Overall, visibility was much worse during the fall experiments, with visibility clear when tidal currents were low, but very poor when the tidal currents were high due to suspended sediment in the water.

The first *M. magister* arrived at the carcasses 6.75 h after submergence and began to feed. A bluntnose sixgill shark, *Hexanchus griseus* (Bonnaterre, 1788), swam over the carcasses on Day 1, but did not feed. It was the only shark observed during both experiments. A piece of tissue was removed from the rump of the exposed carcass on Day 1 but was not witnessed on camera. It may have been due to a shark but its small nature suggests it was crab damage. Crabs were observed feeding at the area on Day 1 (Video S3). Up to six *M. magister* damaged the abdomen of the exposed carcass on Day 1, opening the skin in a 30 cm area, although not breaching the abdominal cavity until Day 2.

The primary fauna on both fall carcasses was *M. magister*, which fed all over the carcasses, not only at the damaged areas (Figure 3). Once the abdomen was breached they would go inside the carcass to feed, sometimes fighting over the resource (Video S4). At times there were ten or more crabs feeding, and at other times no crabs would be present, but their tenure did not correlate with the level of visibility, tide, or oxygen levels. The main site of crab feeding was the abdominal area with most organs removed from the exposed carcass by Day 9. The crabs would lift and move the carcass as they fed (Video S5). Crabs removed much of the tissue from the exposed carcass with the pelvis exposed by Day 13. A few amphipods were observed briefly on occasion at damaged tissue (Table 1), but only in small numbers, and not consistently, until Day 15, when small pools of amphipods appeared on both carcasses on damaged tissue only (Figure 3). They then increased in numbers, spreading over all damaged tissues. The exposed carcass was partially skeletonized by the end of Day 16, with numbers of amphipods fluctuating, sometimes reducing greatly then increasing, and despite removing the majority of soft tissue by Day 19 and complete skeletonization by Day 22, the number of amphipods increased, with the entire cage area thickly covered in roosting amphipods (Video S6), which did not recede until Day 28.

The caged carcass was considerably less damaged by crab feeding when the amphipods first arrived, and they rapidly covered the entire carcass by Day 19, covering both damaged tissue and intact skin (Figure 3). As before, when *S. alascanus* or crabs sloughed amphipods from the carcass, undamaged skin was visible, indicating the amphipods were going into the carcass rather than feeding on the skin directly. The amphipods continued to increase in number, covering the entire platform floor area and ropes and were much more numerous than seen in spring (Video S7). The crabs appeared to avoid the amphipods and when they were present in very large numbers, avoided the carcasses entirely, returning when amphipod numbers receded. The soft tissue on the exposed carcass was rapidly consumed by amphipods, resulting in skeletonization by Day 24. A few crabs returned to feed on bits of loose soft tissue and cartilage (Video S8). The last amphipods left both carcasses on Day 29 several days after skeletonization. At least one, and sometimes several, *S. alascanus* were commonly present, often resting in the cage areas for hours, or even days, at a time, and were present until recovery.

### 3.3. Chemical and Physical Oceanic Measurements

**Figure 4:**
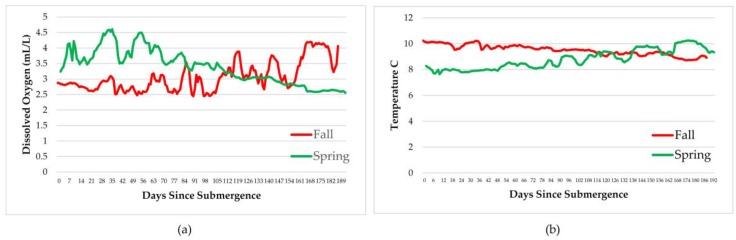
Daily average dissolved oxygen levels (**a**) and temperature (**b**) over the duration of the experiments at a depth of 170 m in the Strait of Georgia in spring and fall (Ocean Network Canada’s VENUS observatory).

**Figure 5:**
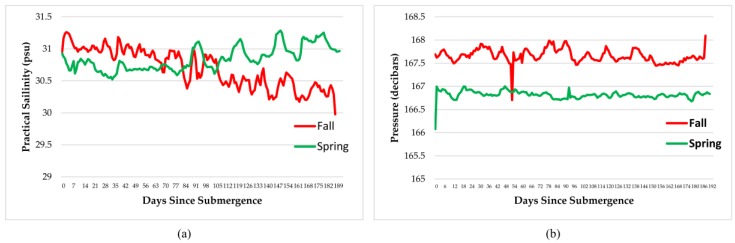
Daily average salinity (**a**) and pressure (**b**) over the duration of the experiments at a depth of 170 m in the Strait of Georgia in spring and fall (Ocean Network Canada’s VENUS observatory).

**Figure 6:**
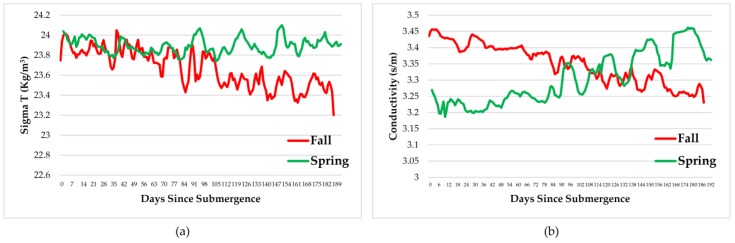
Daily average density as Sigma T (**a**) and conductivity (**b**) over the duration of the experiments at a depth of 170 m in the Strait of Georgia in spring and fall (Ocean Network Canada’s VENUS observatory).

A number of instruments recorded temperature, dissolved oxygen, salinity, pressure, density, and conductivity every minute for the duration of the experiments. These are shown in Figure 4, Figure 5 and Figure 6. As expected, oxygen levels were slightly higher in spring and temperatures slightly lower, probably caused by an influx of cold fresh water from snow melt and rain. This would also impact all of the other parameters. In general, all parameters remained well within the normal ranges for the arthropods involved.

## 4. Discussion

The carcasses deployed in the present study, at 170 m in the Strait of Georgia, were scavenged and skeletonized very differently from those deployed at 300 m in the same Strait. At 300 m, carcasses in both spring and fall were very rapidly covered in Lyssianassidae amphipods [25]. The present spring carcasses also rapidly attracted amphipods, but in much fewer numbers and the fall carcasses did not attract appreciable numbers of amphipods until Day 15. At 300 m, amphipods rapidly increased in numbers on both carcasses and in each season until, by Day 1, all carcasses, as well as the surrounding cage area, were covered in amphipods many animals deep [25]. Numbers continued to increase, with amphipods roosting on all surfaces and pooling onto the substrate surrounding the cage area for at least a metre, and in fall, not only covering the cage floor, edges, and ropes, but also completely covering the bars over the carcass briefly. The carcasses at 300 m began to visibly lose biomass by Day 2, with the fall carcasses skeletonized on Day 3, and spring carcasses on Day 4 [25].

In the present experiments at 170 m, in spring, although amphipods did arrive within hours of placement, they were in much fewer numbers and never reached the density seen at 300 m. In fall, only very few amphipods were present in the first two weeks. The reduction in the numbers of amphipods in both spring and fall at 170 m, meant that other, larger, crustaceans were able to feed on the carcasses. At 300 m, although *P. platyceros* were attracted to the spring carcasses immediately, they were rapidly excluded by the increasing numbers of amphipods, and did not return until the amphipods had receded and in fall, both *P. platyceros* and *M. magister* did not attend the carcasses until after the amphipods were gone. This meant that the 300 m carcasses were almost entirely skeletonized and disarticulated by the actions of the amphipods alone [25]. In the present experiments, with fewer amphipods in spring and almost none in fall for the first two weeks, the larger crustaceans played a much greater role in skeletonizing the carcasses. In spring, *P. platyceros* fed on the carcasses in large numbers, mostly avoiding areas with large masses of amphipods, so feeding primarily around the edges of the carcasses, shredding the skin and tissue. Almost no shrimp were present on the fall carcasses.

Although very few *M. magister* were present in spring (and those observed were small), in fall, the major scavenger was *M. magister* for the first two weeks. The large crabs were attracted immediately and ripped open the carcass and fed on all areas of the carcasses. They fed particularly on the exposed carcass which may have been a function of the cage bars which were designed to prevent shark’s from feeding. The bars were wide enough to allow large crabs to access the caged carcass and many did, but the bars may have proven a deterrent. The exposed carcass had consequently lost much of its soft tissue before amphipods began to arrive in larger numbers.

In spring at 170 m, and in both seasons at 300 m, amphipods covered the entire carcasses, but in both sets of experiments, they did not feed on the skin at first. When fish sloughed amphipods from the carcasses, undamaged skin, hair and even silt on the skin could be seen, indicating that the amphipods entered the carcasses through orifices and openings and fed internally and many of those observed were just roosting. This phenomenon is also observed by recovery divers in which human bodies in dive suits are entirely covered in roosting amphipods and, when recovered, amphipods are seen to emerge from orifices over many hours [26]. When tissue is damaged, as it was by crabs in the fall experiments, the amphipods preferentially covered the areas of exposed muscle tissue and did not cover the areas of remaining skin at first. This was also seen in experiments in nearby Saanich Inlet at 100 m, where pig carcasses were first scavenged by sharks, then *M. magister* and *P. platyceros* [12]. Although much fewer amphipods were observed and did not appear until Day 17, they covered only exposed flesh, feeding under the skin. The skin itself was consumed by numerous squat lobsters, *Munida quadrispina* Benedict, 1902 [12].

Amphipods have been shown to eat pig skin, as they were the only scavengers at 300 m, completely skeletonizing the carcass, eating all the soft tissue [25], but it is clearly a less desired food substance. Previous experiments have shown that amphipods preferentially feed on muscle and fat and can differentiate between food types and texture [27]. In experiments in which porpoise and dolphin carcasses were submerged at depths of 4000–4800 m in the Atlantic Ocean, the carcasses were skeletonized by a succession of amphipod species within six days, although the skin was not eaten [28]. Observations of a decomposing Pilot whale (*Globiecephela mela* Traill, 1809) and an intact Minke whale (*Balaenoptera acutorostrata* Lacépède, 1804) deployed at 30 m and 125 m in a Swedish fjord showed that the bulk of the soft tissue was removed by Atlantic hagfish (*Myxine glutinosa* L., 1758), sharks and other scavengers and amphipods (*O. obtusa*) were not attracted until the skin was open and bones exposed [29]. They were then present in dense populations, growing and reproducing on the remaining tissue from five weeks to four months after submersion, although images indicate that they were not present in the densities observed here in the Strait of Georgia at either 300 m or in spring at 170 m [25,29] and is closer to that seen in Saanich Inlet, when amphipods were only present after large wounds had been opened [12]. In these experiments, and those at 300 m, the amphipods clearly avoided the skin until it was the last soft tissue remaining, when it was finally consumed.

Large openings in the skin had little impact on the time it took amphipods to skeletonize carcasses. In the earlier experiments at 300 m, Bluntnose sixgill sharks took large bites from the exposed carcass and the caged carcass did not display any damage, yet both exposed and caged carcasses were skeletonized at the same time [25], showing that openings in the skin did not accelerate the amphipods’ ability to enter the carcasses. In these fall experiments, only a few amphipods were present from Day 8, with much larger numbers appearing on both carcasses on Day 16. By this time, much of the exposed carcass biomass had been removed by *M. magister* and the caged carcass was still mostly intact. Despite these differences, when the amphipods arrived in large numbers, both carcasses were skeletonized within 24 h of each other, showing the exceptional feeding efficiency of the amphipods’ in comparison with that of the large crabs.

A disadvantage of remote monitoring is that it is not possible to collect samples of fauna, so the actual species of amphipods are not known, although earlier sampling from this area suggests the dominant species is *Orchomenella* aff. *obtusa* [25]. However, it is possible that more than one species fed. Reports of amphipods in the *Orchomene* group have suggested that some species are nocturnal [30,31,32], however, in both of these experiments, and those at 300 m [25], once present, the amphipods did not exhibit any diel activity, although at 170 m, the numbers of amphipods did appear to wax and wane, but this did not appear to follow a diel pattern and may have been more influenced by tides and currents.

As there were fewer amphipods in spring at 170 m and amphipods were delayed in fall, the scavenging patterns were quite different from those seen when only amphipods were feeding. When only amphipods were present, carcasses appeared to ‘deflate’, losing biomass and gradually collapsing under the amphipods, which then receded to reveal complete skeletonization. This occurred at 300 m [25] and in the caged carcass in fall in these experiments. In contrast, with fewer amphipods present, the larger crustaceans picked at the skin and inner soft tissue with chelicerae, shredding the skin, with small pieces drifting in the water current. This presented a very different taphonomic pattern. Additionally, at 300 m, once the carcasses were skeletonized, the majority of the amphipods dispersed, with very low numbers returning briefly [25]. However, in both spring and fall at 170 m, amphipods remained in very large numbers for some days after skeletonization.

The exclusion of the larger crustaceans by large masses of amphipods and the repeatedly observed avoidance of amphipods by *P. platyceros* and *M. magister* could suggest that the smaller crustaceans were competitively excluding the larger crustaceans, or simply proved physically irritating. However, in the experiments at 300 m in the Strait of Georgia, optodes were placed very close to the carcasses in one deployment and it was shown that extremely large numbers of amphipods caused a dramatic, but very localized, drawdown of oxygen [25]. The experiment was repeated to confirm this result and a similar drawdown was noted [33]. In experiments which exposed *Orchomene* sp. to bait odors, increased respiration rates and elevated oxygen consumption was recorded for up to eight hours [34], so it is very probable that the amphipods create a localized anoxia, repelling larger crustaceans. This is unlikely to affect the feeding amphipods as *O. obtusa* has been experimentally shown to survive 10–33 h of anoxia [35]. In these experiments, the optodes were not close enough to the carcasses to measure a localized drawdown and the numbers of amphipods were much fewer than those seen at 300 m, so may not have produced the same levels of hypoxia. This may be why the larger crustaceans remained at the carcasses and fed around the edges of the amphipod masses.

Measurements of dissolved oxygen, temperature, salinity, pressure, density, and conductivity varied over the time period and with season but remained within normal ranges for the species involved therefore, had little impact on the faunal scavenging, unlike that seen in Saanich Inlet, where dissolved oxygen levels drove the scavenging [12].

Very few fish were associated with the carcasses. At 300 m in this Strait, bluntnose sixgill sharks were rapidly attracted to the spring carcasses in both a test run, in which a single deployed carcass was entirely consumed by a number of sharks, and in the actual experiments, where large pieces of tissue were removed by several sharks in the first 24 h after deployment until large masses of amphipods covered the carcasses [25]. However, at 170 m, sharks did not feed on any of the carcasses and only one was seen in the vicinity briefly in fall. At 100 m in Saanich Inlet, on two single occasions, a shark removed part of the carcasses in fall [12]. The bluntnose sixgill shark is typically found in deep waters with a few adults and more commonly, juveniles, in shallower waters [36]. The Strait of Georgia is part of this very widely-distributed demersal species’ range, but local research using time lapse cameras has shown that this species is mostly present in the Strait in summer, with numbers increasing in June and peaking from mid-June to mid-July, and no specimens observed between October and May [37]. These carcasses were deployed in March and late September so the spring carcasses would have already been skeletonized by the summer and the fall carcasses were deployed just at the end of this range. However, the spring carcasses at 300 m, as well as the preliminary test carcass were deployed in February and all attracted several sharks, so obviously some sharks are still present at this time of year, albeit perhaps in much lower numbers.

More shortspine thornyhead fish (*S. alascanus*) were present in spring than in fall, and this was also observed at 300 m in Strait of Georgia [25], in both these and the previous experiments, they frequently rested on the seabed for hours or days at a time, which is typical of these rockfish [38]. However, in the experiments at 300 m, the rockfish seemed to just rest near the carcasses, but did not feed either on the invertebrates on the carcasses or on the carcasses themselves [25]. In the present experiments, the rockfish did mostly rest near the carcasses, but on several occasions they were observed to attempt to catch a shrimp and were seen to feed on the carcass, although rarely, and at one point picked up a mouthful of bones and removed them from camera view (Video S2). It is surprising they did not feed more on the arthropods as although their diet more commonly includes cephalopods, echinoderms, and copepods, they do eat some arthropods [39]. At 300 m the carcasses were disarticulated almost entirely by the amphipods, but at 170 m, the spring carcasses remained articulated after skeletonization by both amphipods and shrimp, and were disarticulated by the action of these fish.

As was observed at 300 m in Strait of Georgia [25] and in Saanich Inlet [12], the carcasses were scavenged and consumed rather than progressing through the expected aquatic decomposition stages seen in other areas such as nearby Howe Sound [11,40], as well as Puget Sound in Washington State [13], California’s Monterey Bay [18], and in much warmer waters near Italy [41]. However, in those experiments, the carcasses floated for a period of time due to bloat and, in the human case studies, the bodies were recovered after being washed ashore, thus, presumably had also floated for a period of time. In the experiments reported here the carcasses were at too great a depth for bloat to occur [26], thus remaining in contact with the seafloor at all times, making them much more accessible to non-swimming or more weakly swimming arthropods.

The data generated here and in the previous series of experiments are valuable as they provide experimental evidence of the fate of mammalian carcasses (as human proxies) on the seafloor under these conditions. Most previous work relates to cadavers recovered from shallow waters or washed ashore which means that the body was floating in the water column. The majority of experimental or observational data from carcasses on the seabed pertains to extremely large carcasses, such as whale falls, which are not forensically valid proxies for human bodies. These data will be very valuable for recovery divers, coast guard, police, forensic pathologists, coroners and medical examiners in understanding the fate of a body in similar marine environments where recovery decisions need to be assessed.

Past case studies indicate the importance of understanding the impact of marine submergence. A body was recovered in the Gulf of Maine by a trawler after having been submerged for over 32 years. However, there was a distinct difference between the decomposition of the upper part of the body and the lower half which suggested that the two contiguous parts of the body had been exposed to different environments [15]. The upper body was highly degraded, with the majority of bones lost and octopus eggs within the flight suit, yet the lower part of the body was well preserved, which led the authors to conclude that the upper part of the body had been in a well-oxygenated environment and the lower part was well preserved due to having been exposed to an anoxic environment suggestive of burial in silt [15]. This is consistent with what we have earlier observed in low-oxygen environments [12]. In another case, a body was recovered from clam flats at the mouth of the Damariscotta River, where it enters the Gulf of Maine, after being submerged for four weeks. A sea urchin spine and marine animal shell fragments showed that the body had entered the water in the ocean, and had been washed into the flats, and had, therefore, not drowned in the flats [15].

Pigs are often used as human proxies [22], so the results presented here indicate that it is probable that human bodies would be skeletonized in a similar manner in these waters at this depth and these seasons. This is valuable information for family members and recovery divers. Divers need to be aware of what to expect when searching for remains and understand that, at certain times of year, the body might be so thickly covered in amphipods that they may no longer be recognizable as a cadaver.

Although at greater depths in the Strait of Georgia, a cadaver can be expected to be skeletonized in three to four days [25], at 170 m, soft tissue can be expected to remain on the carcasses for much longer. Season also had an impact, with only crab damage at first in fall, before amphipods arrived. Therefore, a body at this depth and in this habitat can be expected to be intact for several days or even weeks, and skin and other tissue might be recovered from the surrounding area. Fewer amphipods were present than at 300 m, but they could still prove a diving hazard, as they can be quite dangerous to divers when in close proximity as they are extremely irritating when they swarm over the divers’ face, biting and attempting to get under the mask and can cause a severe panic reaction and so pose a drowning risk [26]. It is, therefore, important that recovery divers are fore-warned of the expected presence of amphipods and the expected conditions of the remains they are trying to locate.

## 5. Conclusions

Season, depth, and environment impact the taphonomy of a cadaver in the marine environment. Ocean Networks Canada’s VENUS observatory offers an extraordinary opportunity to study carcasses in situ on the seabed. This work, together with earlier experiments, presents insight into the fate of a body in the Salish Sea. It is clear that in hypoxic/anoxic Saanich Inlet, dissolved oxygen levels drive decomposition and scavenging, and that at depths of 100–300 m carcasses are primarily scavenged rather than decompose, such as that seen in much shallower waters [23]. Depth, season, and dissolved oxygen levels impact the major faunal species which are attracted which, in turn, impacts the speed of skeletonization and disarticulation. These data are important to help us understand the fate of a cadaver in the ocean.

## Figures and Tables

**Table 1:** Presence or absence of the major fauna on pig carcasses in spring and fall in Strait of Georgia at 170 m. F = fall, Sp = spring, A = amphipods, *M.m.* = *Metacarcinus magister* (Dana, 1852), *P.p.* = *Pandalus platyceros* Brandt, 1851, *S.a.* = *Sebastolobus alascanus* Bean, 1890. Relative abundance for most species could only be estimated but is given as approximately x = 1–5, xx = 6–25, xxx = 26–100, xxxx = 101–1000, and xxxxx = many 1000s.

Day(s)	A		*M.m.*		*P.p.*		*S.a.*		Other Crabs		Other Shrimp		Gastropods	
	Sp	F	Sp	F	Sp	F	Sp	F	Sp	F	Sp	F	Sp	F
0	xx			x	x				x	x			x	
1	xxxx			xx	x						x		xx	
2	xxxxx		x	xx	xx		x	x	x				x	
3	xxxxx	xx	x	xx	xxx		x		x					
4	xxxxx	x	x	xx	xxx				x					
5	xxxxx		x	xx	xxx		x							
6	xxxxx	x	x	xx	xxx		x		x		x	x	x	
7	xxxxx	x	x	xx	xxx		x		x	x		x		
8	xxxxx	x		xx	xx		x	x	x					
9	xxxxx		x	xx	xx		x	x	x			x		x
10	xxxxx	x		xx	x		x	x						x
11	xxxxx		x	xx	x		x	x	x					x
12	xxxx			xx	xx		x		x					
13–17	xx	xxxx		xx	x	x	x		x		x		xxxx	
18–22		xxxx		x			x	x	x				xxxx	
23–27		xxxx		x			x	x	x				xxxx	
28–35		x					x	x	x			x	xx	
36–55							x		x			x		xxxx
55–end			x				x	x	x			x		xxxx

## Supplementary Materials

The following are available online at www.mdpi.com/2075-4450/8/1/33/s1. Video S1: Day 4, spring. Lyssianassidae amphipods, three spot shrimp (*Pandalus platyceros* Brandt 1851) and Dungeness crab (*Metacarcinus magister* Dana 1852) feeding at a pig carcasses submerged at 170 m in the Strait of Georgia (Ocean Network Canada’s VENUS observatory). Video S2: Day 11, spring. Shortspine thornyhead fish (*Sebastolobus alascanus* Bean, 1890) grabbing pig bones from a carcass at 170 m in the Strait of Georgia (Ocean Network Canada’s VENUS observatory). Video S3: Day 1, fall. Dungeness crab (*Metacarcinus magister* Dana 1852) feeding at rump of a pig carcass at 170 m in the Strait of Georgia (Ocean Network Canada’s VENUS observatory). Video S4: Day 5, fall. Dungeness crabs (*Metacarcinus magister* Dana 1852) fighting over a pig carcass at 170 m in the Strait of Georgia (Ocean Network Canada’s VENUS observatory). Video S5: Day 11, fall. Dungeness crabs (*Metacarcinus magister* Dana 1852) lifting a pig carcass at 170 m in the Strait of Georgia (Ocean Network Canada’s VENUS observatory). Video S6: Day 21 fall. Lyssianassidae amphipods feeding on bones of a pig carcass and roosting on the cage area at 170 m in the Strait of Georgia (Ocean Network Canada’s VENUS observatory). Video S7: Day 21 fall. Lyssianassidae amphipods feeding and roosting on a caged pig carcass and roosting on the cage area at 170 m in the Strait of Georgia (Ocean Network Canada’s VENUS observatory). Video S8: Day 21, fall. Dungeness crabs (*Metacarcinus magister* Dana 1852) moving bones from a pig carcass at 170 m in the Strait of Georgia. Despite skeletonization, large numbers of amphipods remain in the cage area and on the bones (Ocean Network Canada’s VENUS observatory).
